# Determine Corneal Biomechanical Parameters by Finite Element Simulation and Parametric Analysis Based on ORA Measurements

**DOI:** 10.3389/fbioe.2022.862947

**Published:** 2022-04-13

**Authors:** Xiao Qin, Lei Tian, Hui Zhang, Di Zhang, Ying Jie, Hai-Xia Zhang, Lin Li

**Affiliations:** ^1^ Beijing Key Laboratory of Fundamental Research on Biomechanics in Clinical Application, School of Biomedical Engineering, Capital Medical University, Beijing, China; ^2^ Medical Science Research Center, Department of Otolaryngology, Peking Union Medical College Hospital, Shuaifuyuan 1, Dongcheng District, Beijing, China; ^3^ Beijing Institute of Ophthalmology, Beijing Tongren Eye Center, Beijing Tongren Hospital, Capital Medical University, Beijing Ophthalmology & Visual Sciences Key Laboratory, Beijing, China; ^4^ Beijing Advanced Innovation Center for Big Data-Based Precision Medicine, Beihang University and Capital Medical University, Beijing Tongren Hospital, Beijing, China

**Keywords:** ocular response analyzer (ORA), finite element simulation, parametric analysis, corneal biomechanical parameters, ORA output parameters

## Abstract

**Purpose:** The Ocular Response Analyzer (ORA) is one of the most commonly used devices to measure corneal biomechanics *in vivo*. Until now, the relationship between the output parameters and corneal typical biomechanical parameters was not clear. Hence, we defined the output parameters of ORA as ORA output parameters. This study aims to propose a method to determine corneal biomechanical parameters based on ORA measurements by finite element simulation and parametric analysis.

**Methods:** Finite element analysis was used to simulate the mechanics process of ORA measurements with different intraocular pressure (IOP), corneal geometrical parameters and corneal biomechanical parameters. A simplified geometrical optics model was built to simulate the optical process of the measurements to extract ORA output parameters. After that, 70% of the simulated data was used to establish the quantitative relationship between corneal biomechanical parameters and ORA output parameters by parametric analysis and 30% of the simulated data was used to validate the established model. Besides, ten normal subjects were included to evaluate the normal range of corneal biomechanical parameters calculated from ORA.

**Results:** The quantitative relationship between corneal biomechanical parameters and ORA output parameters is established by combining parametric analysis with finite element simulation. The elastic modulus (*E*) and relaxation limit (*G*_∞_) of the ten normal subjects were 0.65 ± 0.07 MPa and 0.26 ± 0.15, respectively.

**Conclusions:** A method was proposed to determine corneal biomechanical parameters based on the results of ORA measurements. The magnitude of the corneal biomechanical parameters calculated according to our method was reasonable.

## Introduction

The cornea is one of the most important refractive media of the eyeball providing 70% ocular refractive power (Hjortdal and Jensen, 1995). The maintenance of corneal refractive function depends on the normal corneal geometry. Abnormal corneal geometry is usually closely related to its biomechanical characteristics (Viswanathan et al., 2015). Therefore, studying corneal biomechanical properties *in vivo* has great significance in diagnosing corneal disease such as keratoconus (Scarcelli et al., 2015; Vellara and Patel, 2015; Elham et al., 2017), individualized surgical design, such as corneal refractive correction (Wang B. et al., 2016; Yildirim et al., 2016; Hwang et al., 2017; Zhang et al., 2017) and corneal cross-linking surgery (Steinberg et al., 2014; Matteoli et al., 2016; Subasinghe et al., 2018).

Ocular Response Analyzer (ORA) and Corneal Visualization Scheimpflug Technology (Corvis ST) are two of the most commonly used devices to evaluate corneal biomechanics in clinic. Both of these two devices assess corneal biomechanical properties based on corneal response under rapid air-puff. Parameters provided by these devices are valuable in diagnosing preliminarily keratoconus (Ayar et al., 2015; Elham et al., 2017; Atalay et al., 2019; Koc et al., 2019). Parameters of these two devices doe not only relate to corneal biomechanics but are also influenced by corneal geometrical parameters and intraocular pressure (IOP) (Wang L.-K. et al., 2016; Vinciguerra et al., 2016; Wu et al., 2016; Nemeth et al., 2017; Herber et al., 2019). The limitation of ORA and Corvis ST in clinical applications make it difficult for researchers to obtain corneal biomechanical parameters to diagnose ocular diseases and evaluate corneal treatment effects. Biomechanically speaking, the morphology of the cornea under the external load depends on its biomechanical properties (Mercatelli et al., 2019), which in turn relies on the inherent properties of corneal tissue. Within the range of the physiological IOP, the cornea is likely a linear elastic and viscoelastic tissue (Zhang et al., 2017; Zhang et al., 2018a; Zhang et al., 2018b), and the corneal biomechanical properties can be determined by the corneal elastic modulus (*E*) and corneal relaxation parameters. We call these parameters “corneal biomechanical parameters”. If the corneal biomechanical parameters can be obtained from these *in vivo* measurements directly, the ORA and Corvis can be used in basic and clinical research more conveniently.

At present, the biomechanical interpretations of ORA output parameters and dynamic corneal response paremeters (DCRs) from Corvis ST have not reached a consensus. Alternatively, appropriate and effective methods to determine corneal biomechanical parameters based on the results of ORA/Corvis ST measurements need to be further explored and verified.

The researchers have explored the mechanical significance of ORA output parameters and DCRs through the following methods: a. analyzing the influencing factors of ORA output parameters and DCRs by *ex vivo* eye globe tests (Bao et al., 2015; Zheng et al., 2016); b. suggesting the correlation between ORA output parameters, DCRs and corneal biomechanical parameters (Glass et al., 2008; Han et al., 2014) based on an ideal simplified model or simulating the process of corneal air-puff test by finite element analysis (Elsheikh et al., 2015); and c. providing new corneal biomechanics-related parameters based on corneal air-puff test (Wang L.-K. et al., 2016; Roberts et al., 2017; Shih et al., 2017; Eliasy et al., 2019). In previous studies, we suggested a method to explore the mechanical interpretation of output parameters of ORA (Qin et al., 2019b) and proposed a method to determine corneal elastic modulus based on Corvis measurements (Qin et al., 2019a). However, an effective method to obtain corneal biomechanical parameters directly from ORA output parameters is still lacking. To this end, the present study proposes a method to determine corneal typical biomechanical parameters from ORA measurements.

Actually, it is very sophisticated and difficult to establish a theoretical formula. It is expensive and impractical to establish this relationship based on a large number of ORA measurements and biomechanical tests of the cornea *in vitro*. A feasible and economical way is finite element simulation with the advantage of calculating various loading conditions with the same model. Finite element analysis is used increasingly in the field of corneal biomechanics research (Elsheikh et al., 2011; Kling et al., 2014; Elsheikh et al., 2015; Lago et al., 2015; Sinha Roy et al., 2015; Jannesari et al., 2019). In this study, finite element analysis was used to simulate the corneal response with different corneal biomechanical parameters, corneal geometrical parameters and intraocular pressures (IOP). Additionally, parametric analysis was applied to establish the relationship between ORA output parameters and corneal biomechanical parameters based on a geometrical optics model that computes the ORA output parameters from finite element calculation results. Besides, ten normal subjects were included to evaluate the normal range of corneal biomechanical parameters calculated from ORA.

## Methods

### Finite Element Simulation of ORA Measurements

**FIGURE 1:**
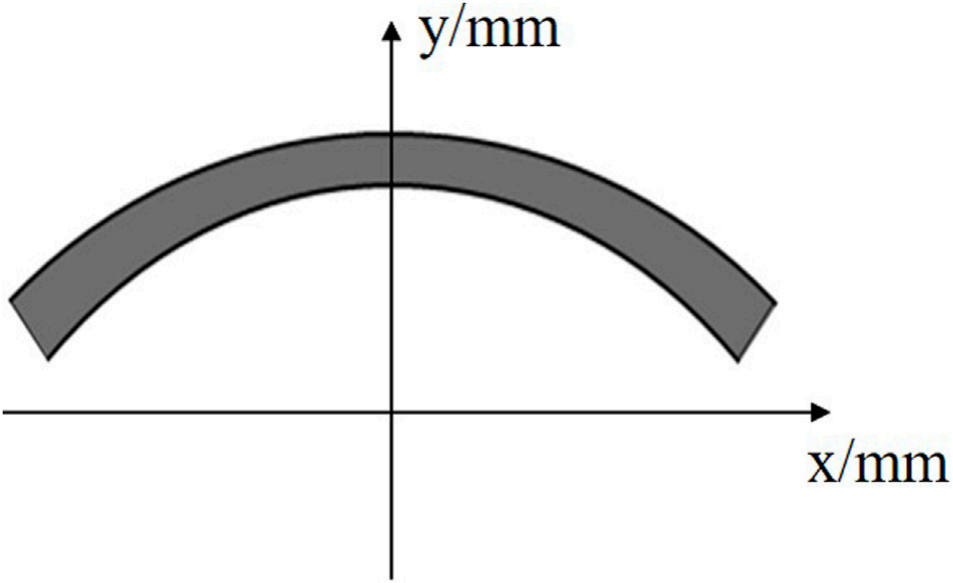
The ideal ellipsoidal axisymmetric corneal geometrical model.

A large number of research results show that both the corneal anterior and posterior surfaces can be described with an elliptic equation (Zhang et al., 2006; Elsheikh, 2010). Therefore, in this study we established an ellipsoidal axisymmetric corneal geometrical model (Figure 1) to carry out dynamic finite element analysis of ORA measurements. The corneal anterior and posterior surfaces can be described as Equation 1 and 2, respectively.

$$\frac{x^{2}}{R^{2}/p}+\frac{y^{2}}{R^{2}/p^{2}}\mathrm{=1}$$ (1)

$$\frac{x^{2}}{a^{2}}+\frac{y^{2}}{{\left(R/\mathrm{p-CCT}\right)}^{2}}\mathrm{=1}$$ (2)

In Eqs. 1, 2, *R* was the corneal central curvature radius, *CCT* was the central corneal thickness, *p* was the ellipse shape factor of the corneal anterior surface. Set *x* to be *R*_0_ in Eqn. 1 we can get the coordinates (*R*_0_, *y*_0_) of anterior corneal limbus. Substitute (*R*_0_, *y*_0_-*PCT*) into Eqn. 2 we can get *a* in Eqn. 2. *R*_0_ was the X coordinate of corneal limbus, *PCT* was the thickness of corneal thickness which was set to be 100 μm larger than *CCT* (Dubbelman, et al., 2009; Elsheikh, et al., 2011; HirjiLarke, 1978).

In the finite element model, the cornea was hypothesized to be linear elastic and viscoelastic material. Corneal elastic modulus (*E*) and Poisson’s ratio (*ν* = 0.49) were used to characterize the corneal linear elastic properties. Third-order Prony model (Eqn. 3) was used to characterize the corneal viscoelastic properties (Qin et al., 2019b). In Eqn. 3, *a*_1_, *a*_2_, *a*_3_, *τ*_1_, *τ*_2_, *τ*_3_ are corneal viscoelastic parameters, *G was the normalized stress during stress relaxation experiment*. As corneal topography is measured at a specific intraocular pressure IOP and is distinct from the unloaded shape that would be obtained at an IOP of 0 mm Hg, the undeformed state was solved by a custom finite element model at first. Air-puff force was applied on corneal apex as a 25 ms surface traction with temporal (Eqn. 4) and spatial (Eqn. 5) normal distribution. Eqn. 4 was obtained by fitting the force-time curve, and Eqn. 5 was obtained by fitting the curve provided by Ref (Elsheikh, 2010). *x* was the distance from the node on the cornea to the corneal symmetry axis. The displacements of limbus are constrained. Cornea was meshed with C3D8R mesh and explicit dynamic analysis was used to simulate the measurements. The finite element analysis was conducted on ABAQUS/Explicit. The variation of corneal anterior surface coordinate along the air-puff force during the measurements was extracted.

$$G\left(t\right)\mathrm{=1-}a_{1}\left(\mathrm{1-}e^{\mathrm{-t}/\tau_{1}}\right)-a_{2}\left(\mathrm{1-}e^{\mathrm{-t}/\tau_{2}}\right)-a_{3}\left(\mathrm{1-}e^{\mathrm{-t}/\tau_{3}}\right)$$ (3)

$$f\left(t\right)=e^{-{\left(\frac{t\left(s\right)\mathrm{-0}\mathrm{.0121}\left(s\right)}{0\mathrm{.0057}\left(s\right)}\right)}^{2}}\left(\mathrm{mN}\right)$$ (4)

$$f\left(x\right)=e^{-{\left(\frac{x\left(\mathrm{mm}\right)}{0\mathrm{.741}\left(\mathrm{mm}\right)}\right)}^{2}}\left(\mathrm{mN}\right)\mathrm{+0}\mathrm{.020}\left(\mathrm{mN}\right)$$ (5)

### Geometrical Optics Simulation of ORA Measurements

**FIGURE 2:**
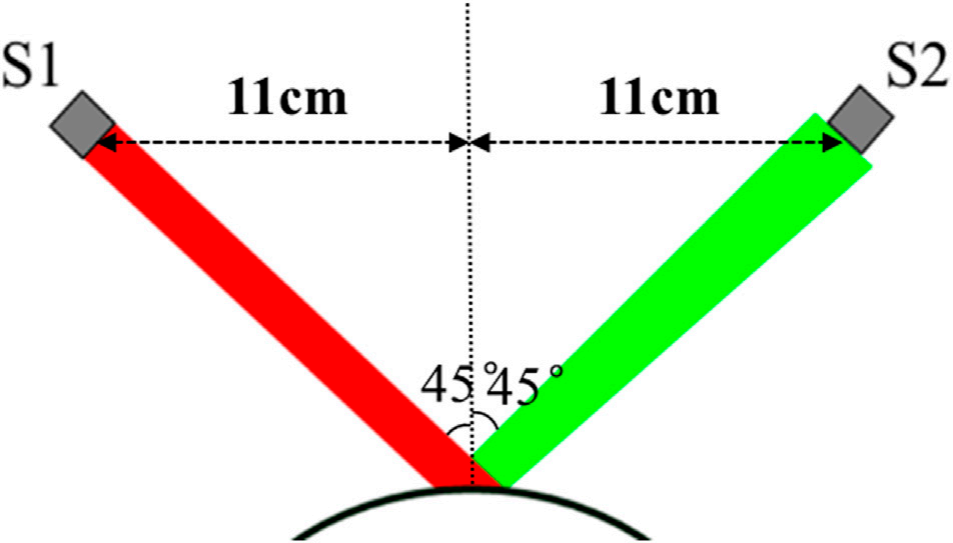
Geometrical optics model of ORA measurement. S1 is a transmitter and S2 is a receiver with a diameter of 3 mm S1 and S2 were on the plane 11 cm away from the corneal apex. The incident angle is 45°. The incident light was reflected by corneal anterior surface and part of the light is received by the receiver S2.

**FIGURE 3:**
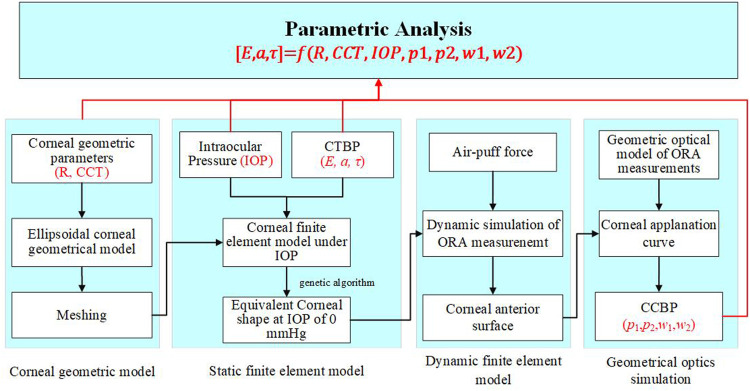
The method to determine corneal biomechanical parameters based on ORA measurement.

According to the principle of ORA measurement, we constructed the ideal geometrical optics model shown in Figure 2. Transmitter S1 and a receiver S2 are on the plane 11 cm away from the corneal apex. The distances between S1 and corneal apex, S2 and corneal apex were both $11\sqrt{2}$ cm. The transmitter emits a parallel incident light (3,000 incident light rays) with a diameter of 3 mm and the incident angle is 45°. The light is reflected by the anterior corneal surface; part of the light is received by the receiver S2 with a diameter of 3 mm. Diffuse reflection from the rough corneal surface and corneal refraction were ignored. Due to the variation of corneal apical position and corneal shape during ORA measurements, the reflected light changes accordingly. According to the ratio of the number of light rays received by the receiver to the total number of incident light rays, we get the normalized light intensity. After that we can obtain the normalized corneal applanation curve which was defined as the variation of the normalized light intensity with time (Figure 3). Based on the normalized corneal applanation curve we can extract the two applanation times *t*_1_, *t*_2_ and the two peak widths *w*_1_ and *w*_2_. And the two applanation pressures *p*_1_ and *p*_2_ can be calculated according to Eqn. 4. *w*_1_, *w*_2_, *p*_1_ and *p*_2_ were used as ORA output parameters in the subsequent parametric analysis.

### Determining Corneal Biomechanical Parameters by Parametric Analysis

Corneal central curvature radius (R), central corneal thickness (CCT) and intraocular pressure (IOP) are important factors affecting ORA measurement results. Therefore, in this study, we set the R, CCT, IOP to be ranged in 6–8 mm, 450–650 μm, 10–30 mmHg, respectively. The corneal biomechanical parameters were mostly reported by biomechanical experiments *in vitro*, such as corneal tensile tests or corneal expansion test. Compared to these *in vitro* experiments, an ORA test was completed within 30 ms, which made there be significant differences between uniaxial tensile test and ORA test in loading mode and magnitude. Corneal non-linear elastic and viscoelastic properties suggest that we adjust the range of corneal biomechanical parameters to make the simulation results consistent with the experimental results. Our previous study (Qin et al., 2019b) found that the simulated Corneal Hysteresis (CH) and Corneal Resistance Factor (CRF) have a similar amplitude with the experimental results when we set the corneal elastic (*E*) to be 1/3 of corneal physiological elastic modulus obtained from the uniaxial tensile test and set the parameters *τ*_1_, *τ*_2_ and *τ*_3_ of third-order Prony series to be 1/10 of the uniaxial tensile test results. According to the reported range of corneal biomechanical parameters, the corneal elastic modulus was varied in the range of 0.2–0.6 MPa (Elsheikh et al., 2008; Shih et al., 2017; Wang et al., 2017; Qin et al., 2019b), *a*_1_ and *τ*_1_ of the third-order Prony series varied in the range of 0.25–0.6 and 0.001–0.1 s, respectively, when we carried out parametric analysis. *a*_2_ and *a*_3_ were set to be 0.1. *τ*_2_, *τ*_3_ were set to be 0.0001 s (Yang et al., 1999).

**FIGURE 4:**
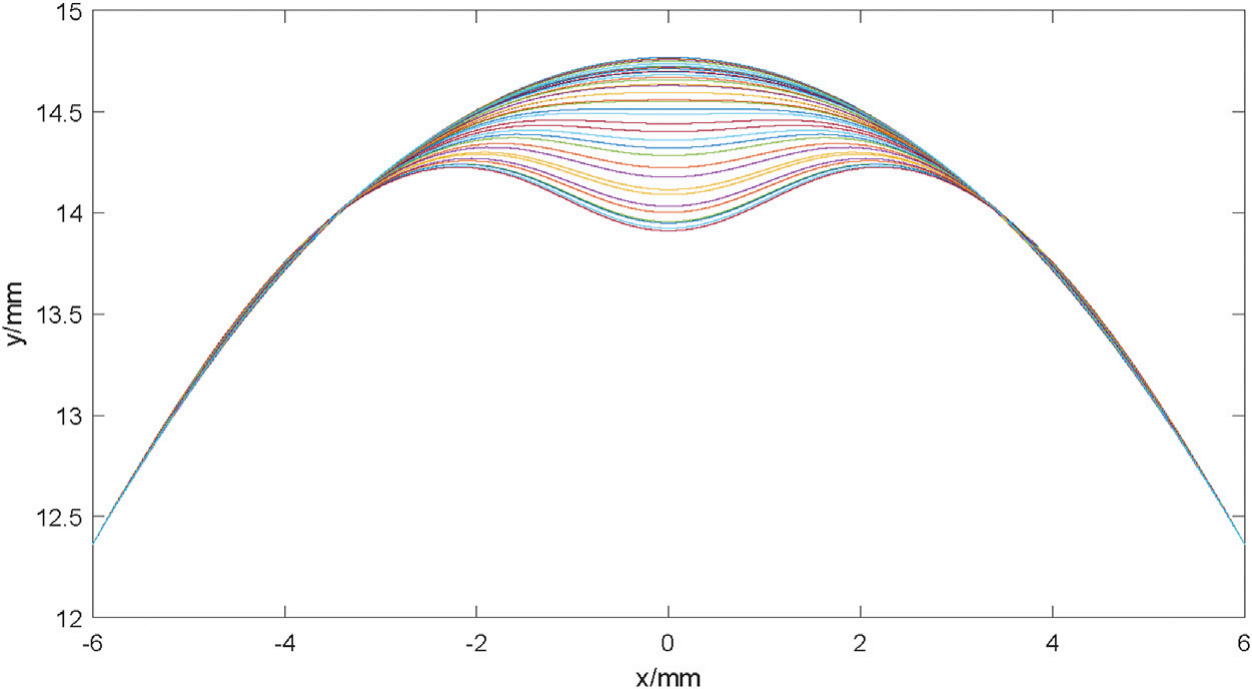
The corneal anterior surface contour at different times during ORA measurements. The cornea gradually deforms from the initial state to the first applanation state, and then to the concave state during the 25 ms measurement. With the air puff pressure removed gradually, the cornea deformed to the initial state gradually.

The flowchart to determine corneal biomechanical parameters based on ORA measurement is shown in Figure 4. For any a given set of parameters R, CCT, IOP, E, *a*_1_ and *τ*_1_, the finite element analysis was used to simulate the corneal response to an air-puff. The normalized corneal applanation curve was obtained by geometrical optic simulation. According to our previous studies and the reported results, the first applanation time (*t*_1_), the second applanation time (*t*_2_), the width at the 50% height of the peak of the first peak (*w*_1_) and second peak (*w*_2_) can reflect corneal biomechanical properties. Therefore, in this study, these four parameters were recorded as ORA output parameters for parametrical analysis.

The produce started with a generation of a random matrix of 2000 × 6 with MATLAB, representing 2,000 times input for 6 parameters (R, CCT, IOP, E, *a*_1_ and *τ*_1_) with uniform distribution in the range of each parameter (shown above). After that, the geometric model of the cornea was constructed, followed a start-up of ABAQUS to fulfill the calculation automatically. The relationship model between corneal biomechanical parameters and ORA output parameters, corneal geometric parameters and IOP was established by multiple quadratic regression model. We took 70% of the data randomly to train the model and the other 30% data were used to verify the established model.

### Subjects and Measurements

Ten healthy subjects (10 eyes) were included in this study. The age of subjects was between 20 and 25 years old. No subject had any eye diseases, history of corneal or eye surgery and systemic diseases affecting their eye functions. All subjects took off soft contact lenses or hard contact lenses at least 1 month before the examination. For each subject, one eye was selected randomly and included in the study. All subjects were informed the consent and had signed the informed consent form before the examination. The informed consent form was in compliance with the tenets of the Declaration of Helsinki. This study was approved by the institutional review board of the Beijing Tongren Hospital, Beijing Institute of Ophthalmology, Beijing, China.

Since the ORA test did not provide corneal geometrical parameters such as CCT and R, this study conducted three ORA tests and one Corvis test for all subjects. All ORA and Corvis measurements were performed by the same technician. During the ORA test, any measurement result with a waveform score (WS) exceeding 3.5 was included. The Corvis test result was included when the reading of “alignment” was “OK”. Otherwise, the measurements were repeated until the reading was “OK”. The edge of the first undeformed corneal image obtained by Corvis test is extracted to obtain the CCT and R (Qin et al., 2019a), and the corneal biomechanical corrected IOP (bIOP) was read from the Corvis test results for the subsequent calculating of corneal biomechanical parameters.

## Results

**FIGURE 5:**
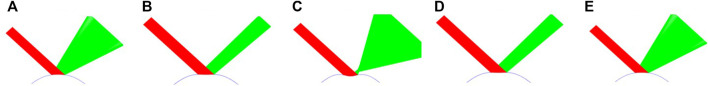
Optical paths at different times during ORA measurements. **(A)** initial state; **(B)** the first applanation state; **(C)** the maximum indentation state; **(D)** the second applanation state; **(E)** the end state.

From the output database of the finite element simulation model of ORA measurements, we obtained the coordinate files of the anterior corneal surface at different times. The profile of the anterior corneal surface at different times during ORA measurements was drawn by using the file reading and writing function, as well as the drawing function of MATLAB for the subsequent geometrical optical simulation. The results are shown in Figure 5.

**FIGURE 6:**
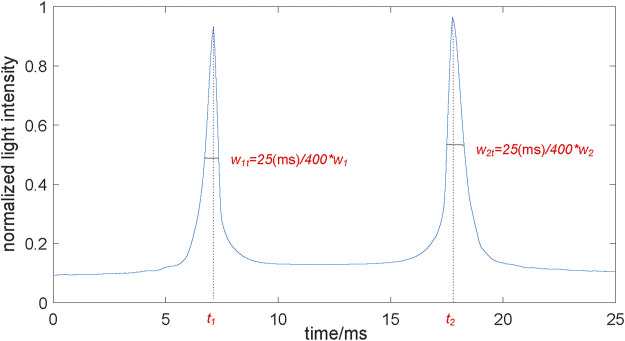
The normalized corneal applanation curve. The normalized lighted intensity was the ratio of the number of light rays received by the receiver to the total number of incident light rays. When the cornea reached the applanation during the load and unload state, the curve reaches at a peak, respectively. *t*_1_, *t*_2_, were the two applanation time. *w*_1t_, *w*_2t_ were the two peak widths, which were proportional to *w*1, *w*2 of the ORA output.

The results of the geometrical optical simulation are shown in Figure 6. Due to the variation of the corneal apical position and the corneal shape during ORA measurements, the reflected light changes accordingly. Figure 6 (a–f) represents the typical optical path at different times during ORA measurements.

According to the ratio of the number of light rays received by the receiver to the total number of incident light rays during the ORA test (Figure 6), the normalized corneal applanation curve was obtained (Figure 3). The two applanation times *t*_1_ and *t*_2_ and the two peak widths *w*_1_ and *w*_2_ can be extracted from the normalized corneal applanation curve. The two applanation pressures *p*_1_ and *p*_2_ can be calculated according to Eqn. 4.

**FIGURE 7:**
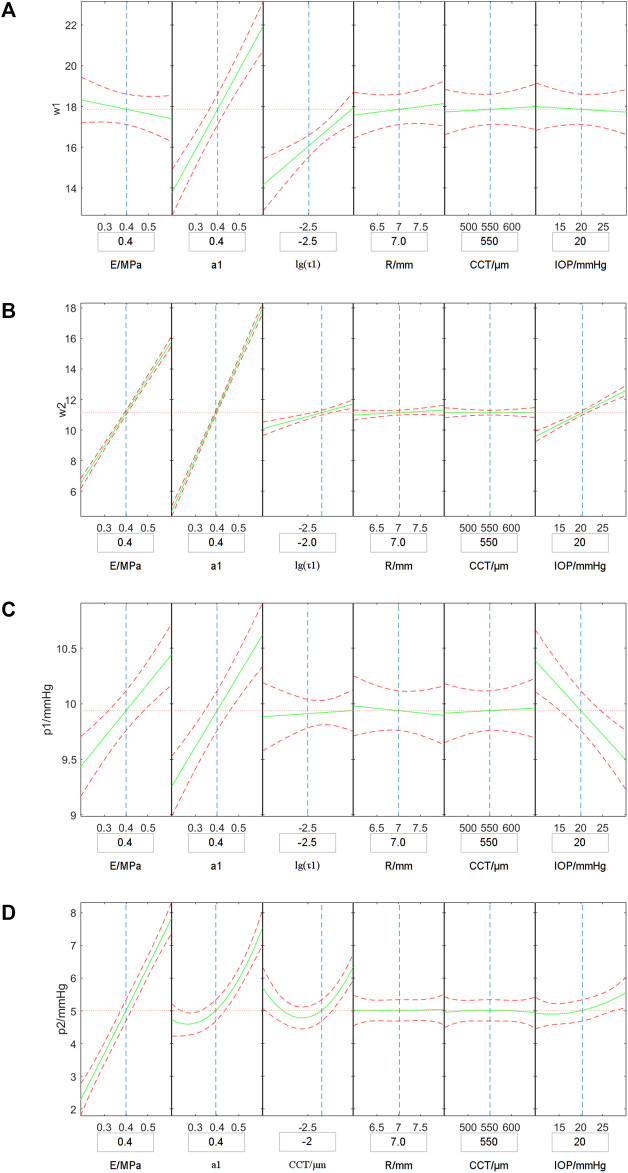
Variations of the simulated *w*_1_ **(A)**, *w*_2_ **(B)**, *p*_1_ **(C)** and *p*_2_ **(D)** with corneal biomechanical parameters, CCT, R and IOP. The vertical axis represents the simulated ORA output parameters and the horizontal axes shows independent variables. The green solid curves simulate the variation of the average simulated ORA output parameters with one independent variable when other parameters set to the value in the boxes under the horizontal axis, and the red dotted curves are the range of the simulated ORA output parameters.

Figures 7A–D shows the variation of the simulated ORA output parameters with corneal biomechanical parameters, CCT, R and IOP. In Figure 7, the vertical axis shows the simulated ORA output parameters while the horizontal axes represent the independent variables. The green solid curves simulate the variation of the average simulated ORA output parameters with one independent variable when other parameters were set to the value in the small boxes below. (i.e., the green curve in the first plot in Figure 7A represented the variation of the simulated w1 with E, when the a1, lgτ1, R, CCT, IOP were set to be 0.4, −2.5, 7.0 mm, 550 μm, 20 mm Hg, respectively). The red dotted curves are the ranges of the simulated ORA output parameters. Results shown in Figure 7 suggest that *w*_2_, *p*_1_ and *p*_2_ are positively correlated with the corneal elastic modulus (*E*) while *w*_1_, *w*_2_, and *p*_1_ are significantly positively correlated with *a*_1_. Furthermore, *w*_1_, *w*_2_ are likely significantly positively correlated with *τ*_1_. Also, *p*_2_ and *a*_1_, *τ*_1_, IOP have a parabolic relationship, while *w*_2_ are positively and *p*_1_ negatively correlated with IOP. The correlations between other parameters were not significant.

The relationship model based on the 70% of the simulated data was established by multiple quadratic regression model. The results are shown in Eqs. 6—8. In the equations, we take the mean value of each parameter (*E*_0_ = 0.4 MPa, *τ*_10_ = 0.001 s, R_0_ = 7 mm, IOP_0_ = 15 mmHg, *w*_10_ = 10, *w*_20_ = 17, *p*_10_ = 10 mmHg, *p*_20_ = 5 mmHg) to make the parameters dimensionless.

E=2.500E0[0.323-0.030 R/R0-0.018 IOP/IOP0-0.034w1/w10-0.078w2/w20+0.230p2/p20] (6)

a1=0.298-0.055 R/R0-0.104IOP/IOP0-0.679w1/w10+0.330w2/w20+0.536p1/p10-0.380p2/p20+0.330(w1/w10)2+0.075(p2/p20)2 (7)

lg(τ1/τ10)=-5.419+0.017 R/R0-0.033 CCT/CCT0-0.013w1/w10+0.140w2/w20-0.086p1/p10-0.165p2/p20 (8)

**FIGURE 8:**
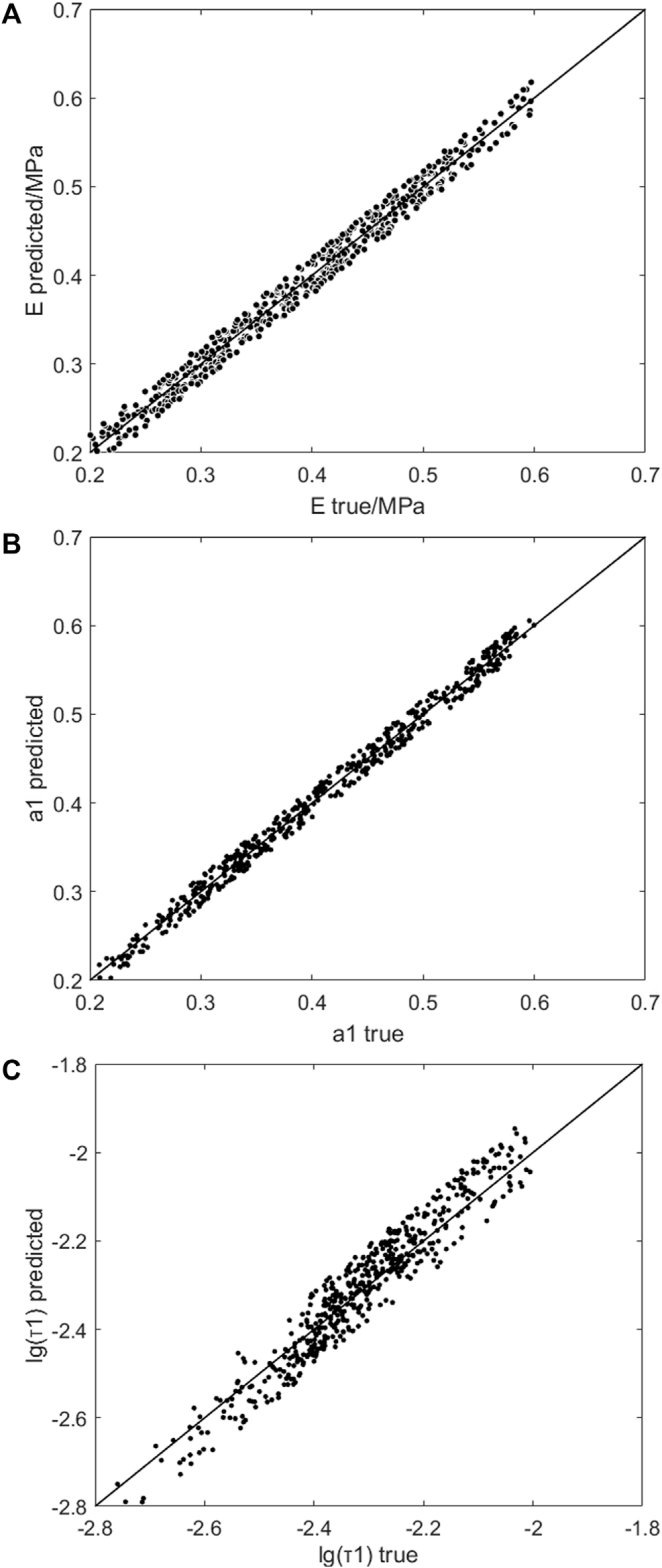
The comparison of the predicted and set *E* **(A)**, *a*_1_ **(B)** and τ_1_ **(C)**. The horizontal axis was the true value in finite element simulation, and the vertical axis was the predicted value of the multiple quadratic regression model.

Figure 8 shows the results of the comparison between the predicted value of corneal biomechanical parameters calculated according to Eqs. 6–8 and the set corneal biomechanical parameters using the remaining 30% data. The results show that there was good consistency for the corneal elastic modulus *E*, corneal viscoelastic parameter *a*_1_, τ_1_ between the predicted value and the true value. This indicates that the multiple regression model might be enough to describe the relationship between corneal biomechanical parameters and ORA output parameters.

For the ten healthy subjects, the CCT, R, and corneal biomechanics corrected intraocular pressure (bIOP) were 526.1 ± 31.1 μm, 7.77 ± 0.47 mm and 18.7 ± 2.4 mmHg, respectively. For each one, *t*_1_, *t*_2_, *w*_1_ and *w*_2_ were extracted from the ORA database. The *p*_1_, *p*_2_ were calculated from *t*_1_, *t*_2_ according to Eqn. 4. The average of the three ORA measurement results was used to calculate the corneal biomechanical parameters The ORA test results are shown in Table 1, which also provides the results of corneal biomechanical parameters calculated according to Eqs. 6–8. We can see that the *a*_1_ of normal subjects were 0.54 ± 0.15, as the *a*_2_ and *a*_3_ were set to 0.1 in the third-order Prony series. The relaxation limit (*G*_∞_ = 1-*a*_1_-*a*_2_-*a*_3_) of the normal subjects were 0.26 ± 0.15. The magnitude of *E* and *G*_∞_ were basically consistent with the results of corneal uniaxial tensile test (Elsheikh et al., 2008; Wang et al., 2017).

**TABLE 1:** Results of ORA measurements and the calculated corneal biomechanical parameters in healthy subjects.

parameters	*p*_1_/mmHg	*p*_2_/mmHg	*w*_1_	*w*_2_	*E*/MPa	*a*_1_	*τ*_1_/s
Mean	17.45	10.67	11.10	15.20	0.65	0.54	0.00313
SD	3.09	1.68	1.52	4.10	0.27	0.15	0.00033

## Discussion

In this study, the dynamic finite element simulation and geometric optical simulation of the ORA measurement process with different geometric parameters (CCT and R), intraocular pressure (IOP) and corneal biomechanical parameters (*E*, *a*_1_ *τ*_1_) were carried out to obtain ORA output parameters. Through a parametric study, we proposed a method to determine the corneal biomechanical parameters based on ORA measurements. The results of these studies are of great significance for the further promotion of ORA in clinical applications.

It is important to determine the range of the parameters in parametric analysis. The range of corneal geometrical parameters and IOP can be get from the reports on corneal Corvis or Pentacam measurements conveniently. The corneal biomechanical parameters were mostly reported by biomechanical experiments *in vitro*, such as corneal tensile tests or corneal expansion test. Compared to these *in vitro* experiments, an ORA test was completed within 30 ms, which made there be significant differences between uniaxial tensile test and ORA test in loading mode and magnitude. Corneal non-linear elastic and viscoelastic properties suggest that we adjust the range of corneal biomechanical parameters to make the simulation results consistent with the experimental results. The results in our previous study showed that when the cornea elastic modulus was set to be 1/3 of the corneal elastic modulus in physiological range obtained by uniaxial tensile test, and τ_1_, τ_2_ and τ_3_ were set to be 1/10 of the corneal stress relaxation results, the amplitudes of ORA output parameters obtained by finite element simulation was basically consistent with the measured values. In this study, we set the *E* to the range of 0.2–0.6 MPa, *a*_1_ and *τ*_1_ of the third-order Prony series varied in the range of 0.25–0.6 and 0.001–0.01 s, respectively, when we carried out parametric analysis referred to these results (Qin et al., 2019b).

Although CH and CRF are two of the comprehensive ORA output parameters obtained directly from the ORA test, which were derived from the linear combination of the two applanation pressure (*p*_1_ and *p*_2_) (Luce, 2005), there is no consistent report on the relationship between CH, CRF and *p*_1_, *p*_2_. Both ORA test and finite element simulation can easily obtain *p*_1_ and *p*_2_, therefore, this study directly used *p*_1_ and *p*_2_ as ORA output parameters for parametric analysis. In geometric optics simulation of ORA test, corneal surface was regarded as smooth surface. The influence of corneal surface roughness, tear film and other factors were ignored. These factors may affect some of the applanation curve waveform parameters such as the applanation peaks height *h*_1_, *h*_2_, etc. Yet, the influence on the width of peaks (*w*_1_ and *w*_2_) were relatively small (Nakao et al., 2017). Our previous study also found that *w*_1_, *w*_2_ were significantly correlated with corneal biomechanical parameters. Therefore, in this study, *w*_1_ and *w*_2_ were used to determine the corneal biomechanical parameters.

Cornea is a nonlinear elastic and viscoelastic biological soft tissue. As the cornea is still within the physiological range under the action of fast air-puff (Wang et al., 2017; Zhang et al., 2018a; Zhang et al., 2018b), the cornea was regarded as linear elastic and viscoelastic material for the finite element simulation in this study. As can be seen in Figure 7, with an increase of corneal elastic modulus (*E*), the simulated values of *p*_1_ and *p*_2_ also increased. Also, with the increase of the viscoelastic parameter *a*1, the parameters *w*_1_, *w*_2_, *p*_1_ and *p*_2_ showed upward trends. With the increase of the viscoelastic parameter *τ*_1_, the simulated values of *w*_1_ and *w*_2_ increased, and the simulated values of *p*_2_ decreased first and then increased. These results indicate that the four ORA test parameters we selected can reflect the biomechanical properties of the cornea.

In addition to the corneal biomechanical parameters, corneal geometric parameters such as central corneal curvature radius (R), central corneal thickness (CCT) and intraocular pressure (IOP) can also affect the ORA measurement results (Terai et al., 2012; Hwang et al., 2013; Sharifipour et al., 2016; Fujishiro et al., 2020). Therefore, this study further examined the influence of these parameters on the finite element simulation results to obtain more accurate corneal biomechanical parameters. The results showed that with the increase of IOP, the simulated values of *w*_2_ and *p*_2_ increased, while the simulated values of *p*_1_ decreased. With the increase of R, the simulated values of *w*_1_ and *w*_2_ increased.

Based on the finite element simulation results of corneal ORA tests with different corneal geometric parameters, IOP, and corneal biomechanical parameters we established a multiple quadratic regression model to determine corneal biomechanical parameters As shown in Eqs. 6–8, *E* was negatively correlated with *w*_1_ and *w*_2_ while being positively correlated with *p*_2_. This, however, was basically consistent with the negative correlation between *E* and *w*_1_, *w*_2_ reported in our previous study (Qin et al., 2019a). As high IOP and R will lead to overestimation of corneal elastic modulus, the coefficients of IOP and R in Eqn. 6 were negative, thus weakening the influence of intraocular pressure and corneal radius of curvature on the calculation results of *E*. According to Eqs. 7, 8, the viscoelastic parameter *a*_1_ has a postive correlation with *w*_2_, *p*_1_ and a nonlinear relationship with *w*_1_ and *p*_2_. The viscoelastic parameter *τ*_1_ is negatively correlated with *w*_1_, *p*_1_ and *p*_2_ while being positively correlated with *w*_*2*_. If one disregarded the differences of IOP and R, the viscoelastic parameter *a*_1_ of subjects with high IOP and R would be overestimated.

The amplitude of air-puff pressure provided by ORA varied among different subjects according to corneal conditions. However, the pattern of air-puff amplitude provided by ORA has not been reported. In this study, the finite element simulation of ORA test ignored the difference of air-puff pressure, and the obtained corneal elastic modulus *E* ranged from 0.3 to 0.8 MPa, which was basically consistent with the order of magnitude of human corneal elastic modulus reported in literature (Wang et al., 2017). The viscoelastic parameters *a*_1_ and *τ*_1_ ranged from 0.3–0.6 and 0.002–0.005 s, respectively. Since both *a*_2_ and *a*_3_ were set to be 0.1 in the third-order Prony series, the relaxation limit (*G*_∞_) ranged from 0.2 to 0.5, which was basically consistent with the results of corneal uniaxial tensile test (Elsheikh et al., 2008). In addition, it can be seen in Figure 8 that the CTBP calculated by Eqs 6–8 are in good consistency with the corneal classical biomechanical parameters input in finite element simulation. Besides, there was a good consistency between the corneal elastic modulus *E*, *a*_1_, τ_1_ between the predicted value and the set value (Figure 8). These results reflect the validity of the proposed method for determining corneal biomechanical parameters.

One of the limitations was that when geometric optics simulation, the corneal surface was regarded as a smooth surface, influence of corneal surface roughness, tear film and other factors was ignored. Comparing the applanation curves obtained from ORA test and optical simulation, these factors may affect the peak heights *h*_1_ and *h*_2_ in the waveform parameters, and have relatively little effect on the peak widths *w*_1_ and *w*_2_. Therefore, *w*_1_ and *w*_2_ were extracted for analysis. A more accurate optical model considering the refraction and reflection of the tear film on cornea and the unsmooth corneal surface may be established, which will help us obtain more real waveform parameters and establish a more accurate quantitative relationship between corneal biomechanical parameters and ORA output parameters. Another limitation was that the sample size was limited, future studies should be conducted based on a larger number of clinical data for better clinical application. Besides, the finite element simulation of ORA test ignored the difference of air-puff pressure as the pattern of air-puff amplitude provided by ORA has not been reported. According to our previous study (Qin et al., 2019b), the simulated CH and CRF have same magnitude with the experimental results although we have ignored the variation of air-puff amplitude. This reminded us the influence of ignoring the air-puff pressure difference may be ignored.

In conclusion, this study provides a method to determine linear elastic and viscoelastic material parameters of human cornea based on ORA measurements. The corneal biomechanical parameters identified by the present method need to be verified further with a great number of data. The clinic applications of this method we shall also explore, such as in diagnosis of keratoconus.

## Data Availability

The original contributions presented in the study are included in the article/Supplementary Material, further inquiries can be directed to the corresponding authors.
